# Association between HDL-C and depression in U.S. adults: A cross-sectional analysis of NHANES

**DOI:** 10.1097/MD.0000000000045754

**Published:** 2025-11-14

**Authors:** Jing Zheng, Juan Qian, Minhua Li, Qi Wang

**Affiliations:** a Department of Thyroid and Breast Surgery, Affiliated Hospital of Jiangnan University, Wuxi, Jiangsu Province, China; b Department of Radiation Oncology, Affiliated Hospital of Jiangnan University, Wuxi, Jiangsu Province, China.

**Keywords:** cross-sectional study, depression, HDL-C, NHANES

## Abstract

High-density lipoprotein cholesterol (HDL-C) has been increasingly recognized not only for its established role in cardiovascular health but also for its potential relevance to mental health, particularly depression. Dysregulated lipid metabolism may affect neuroinflammation, oxidative stress, and hypothalamic–pituitary–adrenal axis regulation, thereby influencing mood. This study aimed to investigate the association between HDL-C levels and depression in a nationally representative U.S. population. We analyzed cross-sectional data from 12,310 adults in the National Health and Nutrition Examination Survey (2015–2020). Depression was present in 8.8% of participants. Weighted multivariable logistic regression and generalized additive models with restricted cubic spline regression were applied to examine linear and nonlinear associations, and subgroup analyses were conducted. Models were adjusted for key covariates, including age, sex, race/ethnicity, body mass index (BMI), poverty-income ratio, smoking, alcohol use, and comorbidities such as diabetes, hypertension, and cardiovascular disease. Participants with depression had significantly lower mean HDL-C levels than those without depression (weighted mean: 1.37 vs 1.41 mmol/L, *P* < .01). Higher HDL-C levels were inversely associated with depression (OR = 0.92, 95% confidence interval (CI): 0.71–1.13). Subgroup analyses indicated a stronger inverse association among men (OR = 1.92; 95% CI: 0.45–3.39) and in participants without hypertension (OR = –3.64; 95% CI:–5.21 to–2.07). A nonlinear relationship between HDL-C and depression was also observed. HDL-C levels were inversely and nonlinearly associated with depression, with particularly strong associations in men and individuals without hypertension. These findings suggest HDL-C may serve as a potentially modifiable biomarker for depression risk, highlighting the need for longitudinal studies to confirm causality and to evaluate whether lipid management strategies could be incorporated into preventive or therapeutic approaches in neuropsychiatry.

## 1. Introduction

Depression is a prevalent mental disorder characterized by a constellation of symptoms, including persistent low mood, diminished interest or pleasure in daily activities, changes in appetite and sleep, fatigue, difficulty concentrating, feelings of worthlessness or excessive guilt, psychomotor agitation or retardation, and recurrent thoughts of death or suicidal ideation.^[1,2]^ Globally, depression affects more than 280 million individuals, and the World Health Organization projects it will become the leading cause of disease burden by 2030.^[3,4]^ Beyond its psychological toll, depression is associated with substantial physical comorbidities and heightened mortality risk, underscoring the urgency of identifying modifiable biological risk factors that may aid in prevention and early intervention.

High-density lipoprotein cholesterol (HDL-C) is a lipoprotein class responsible for reverse cholesterol transport and has long been recognized for its protective role in cardiovascular disease (CVD) through anti-inflammatory, antioxidative, and endothelial-stabilizing mechanisms.^[5,6]^ Importantly, emerging evidence suggests that HDL-C may also be implicated in mental health conditions, including depression. For example, some studies have reported that low HDL-C levels are associated with an increased risk and prolonged duration of depressive symptoms, whereas others have found opposite or null associations.^[7–12]^ Such inconsistencies may be attributed to variations in study design, population characteristics, and analytic approaches.

From a mechanistic perspective, HDL-C has been proposed to influence depression through several biological pathways. These include modulation of neuroinflammation, regulation of oxidative stress, effects on the hypothalamic–pituitary–adrenal (HPA) axis, and impacts on neurotransmitter function.^[13,14]^ Depression has increasingly been conceptualized as an inflammatory disorder, and HDL-C’s anti-inflammatory properties could plausibly mediate its association with mood regulation.^[15–17]^ Moreover, CVDs, where HDL-C plays a central role, frequently co-occur with depression^[18]^; however, the relevance of cardiovascular findings should be interpreted specifically in relation to how vascular and metabolic disturbances may alter depression risk, rather than as a general discussion of CVD.^[19]^

Despite these insights, there remain significant gaps in the literature. Prior studies have often been limited by small sample sizes, inconsistent adjustment for confounding variables, and lack of exploration of potential nonlinear or subgroup-specific effects. In particular, the dose-response relationship between HDL-C and depression, and whether it varies by sex, body mass index (BMI), or comorbid hypertension, remains unclear.

To address these limitations, we conducted a cross-sectional analysis using data from the National Health and Nutrition Examination Survey (NHANES, 2015–2020). We hypothesized that HDL-C levels are associated with depression in a nonlinear manner and that this association may differ across demographic and clinical subgroups. Our study aims to clarify the putative role of HDL-C in depression and contribute evidence to inform lipid management strategies for improving mental health outcomes.

## 2. Materials and methods

### 2.1. Study population

**Figure 1.**
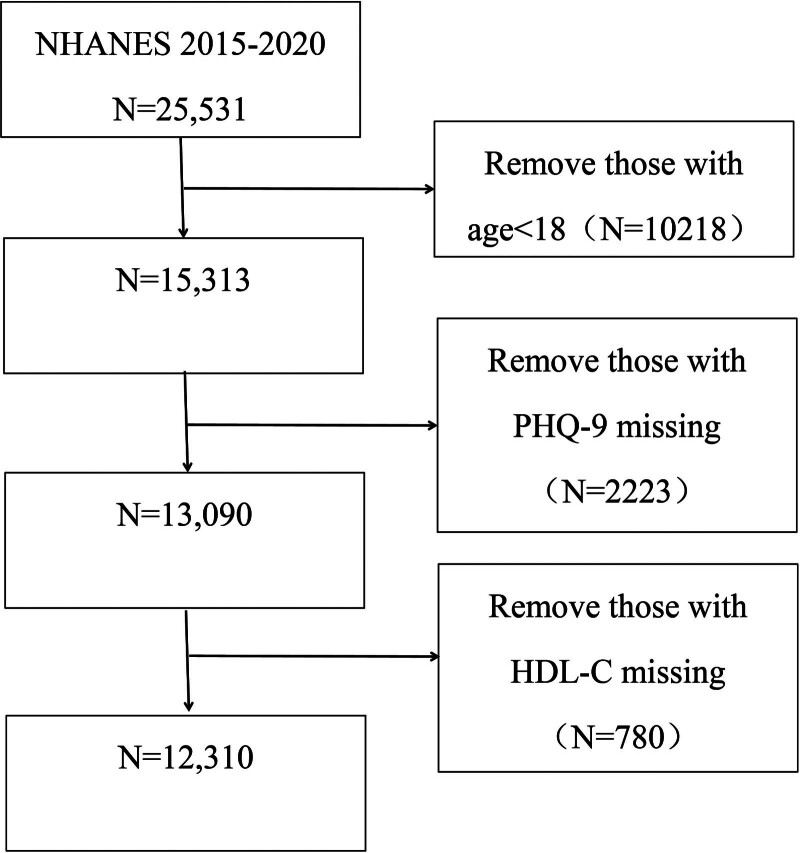
Flow diagram of the inclusion criteria and exclusion criteria.

The NHANES is a nationally representative survey of the U.S. non-institutionalized civilian population that combines in-home interviews with standardized examinations in mobile examination centers. Details of sampling, data collection, and ethics oversight by the National Center for Health Statistics Institutional Review Board are available at the NHANES website (https://www.cdc.gov/nchs/nhanes/). For this analysis, we used NHANES 2015 to 2020 data and included adults aged ≥ 18 years with available HDL-C and depression assessments. We excluded participants missing HDL-C, missing the patient health questionnaire-9 (PHQ-9), or aged < 18 years. After exclusions, 12,310 participants remained (see Fig. 1 for the flow diagram).

### 2.2. Depression

Depression was assessed using PHQ-9, a validated 9-item self-report instrument for depressive symptom severity over the prior 2 weeks. Consistent with standard practice, depression was defined as PHQ-9 score ≥ 10 (moderate-to-severe).^[20]^ Trained personnel administered the PHQ-9 at mobile examination centers following NHANES protocols.

### 2.3. Measurement of serum lipid profiles

Serum lipids, including HDL-C, low-density lipoprotein cholesterol (LDL-C), and triglycerides (TGs), were measured using standardized enzymatic assays per NHANES laboratory procedures. Reference ranges commonly used in U.S. clinical practice are HDL-C ≥ 1.04 mmol/L (40 mg/dL), LDL-C < 3.36 mmol/L (130 mg/dL), and TG < 3.88 mmol/L (150 mg/dL).

### 2.4. Covariates

We meticulously evaluated a wide array of potential confounding factors. These included gender, age, racial and ethnic identity, poverty-income ratio (PIR), the presence of diabetes mellitus, BMI for assessing adiposity, educational qualification level, marital standing, patterns of alcohol consumption, a previous medical history of CVD–encompassing heart failure, angina pectoris, myocardial infarction, and stroke events, smoking status, LDL-C, and TG. In this study, participants were segregated based on multiple sociodemographic and health-related parameters. For race and ethnicity, they were allocated into groups such as non-Hispanic White, Mexican American, non-Hispanic Black, and other ethnic categories. Marital status, educational attainment, smoking habits, and alcohol intake patterns were also classified systematically for further analysis.

### 2.5. Statistical analyses

Analyses were performed using R version 4.3.0 (R Foundation for Statistical Computing, Vienna, Austria; https://www.r-project.org/) and EmpowerStats (X&Y Solutions, Inc.; https://www.empowerstats.com/). All estimates incorporated the six-year NHANES sampling weights and complex survey design. Continuous variables are summarized as means with standard error of the mean; categorical variables as weighted percentages.

We fit survey-weighted multivariable logistic regression models to estimate odds ratios (ORs) and 95% confidence intervals (CIs) for depression across HDL-C quartiles (lowest quartile as reference) and per-unit HDL-C.

Prior to weighted multivariable logistic regression, we assessed key assumptions. Linearity of the logit: For continuous predictors (HDL-C, age, BMI, PIR, LDL, TGs), we conducted Box–Tidwell tests by adding xlogx terms and inspected partial plots of the logit. The Box–Tidwell test indicated nonlinearity for these predictors (Table S1A, Supplemental Digital Content, https://links.lww.com/MD/Q615). Accordingly, HDL-C was additionally modeled using smooth functions (GAM/restricted cubic splines) as reported in the main text; categorization or flexible terms for other continuous covariates are provided in supplementary sensitivity notes; Multicollinearity: We computed variance inflation factors (VIFs) using a design matrix with reference categories removed. Several predictors (BMI, age, HDL-C, LDL) showed elevated VIFs (approximately 17.4, 16.9, 13.6, and 10.1, respectively), reflecting correlation among anthropometric and lipid variables; full VIFs are reported in Table S1B (Supplemental Digital Content, https://links.lww.com/MD/Q615); and Outliers and influence: We examined standardized residuals, leverage, and Cook distance from a weighted binomial generalized linear model. To stabilize influence metrics under complex survey weights, weights were normalized to mean 1 for this diagnostic only (no effect on point estimates). Counts of flagged observations are summarized in Table S1C (Supplemental Digital Content, https://links.lww.com/MD/Q615). No obvious data-entry errors were identified on inspection, and primary analyses retained all observations.

The weighted analyses followed NHANES analytic guidelines. Specifically, we applied the MEC examination weights (WTMEC2YR), which incorporate sampling probabilities and adjustments for non-response, to account for the complex, multistage probability sampling design. When combining multiple survey cycles, weights were divided by the number of cycles to obtain the correct multi-cycle weights, as recommended in the NHANES analytic guidelines. All regression models were estimated using these analytic weights.

Differences in baseline characteristics between depression and non-depression groups, as reported in Table 1, were assessed using weighted χ^2^ tests for categorical variables and weighted *t*-tests for continuous variables. Imbalances across groups were addressed by including all these covariates as adjustment factors in the multivariable regression models, ensuring that estimates for the association between HDL-C and depression were not confounded by differences in baseline characteristics.

**Table 1:** Weighted baseline characteristics of patients with or without depression.

Variable	Total	Without depression	With depression	*P* value
Age (yr)	48.01 ± 17.38	48.12 ± 17.41	46.76 ± 17.02	.02
PIR	3.04 ± 1.57	3.10 ± 1.56	2.30 ± 1.52	<.01
LDL (mmol/L)	2.85 ± 0.63	2.85 ± 0.62	2.88 ± 0.68	.16
HDL-C (mmol/L)	1.41 ± 0.45	1.41 ± 0.45	1.37 ± 0.44	<.01
Triglyceride (mmol/L)	1.26 ± 0.68	1.25 ± 0.67	1.32 ± 0.71	<.01
BMI (kg/m^2^)	29.63 ± 7.07	29.49 ± 6.94	31.22 ± 8.26	<.01
Sex				
Male	48.73	49.67	37.78	<.01
Female	51.27	50.33	62.22	
Race				
Mexican American	8.8	8.91	7.59	.08
Other Hispanic	6.82	6.65	8.73	
Non-Hispanic White	64.79	64.91	63.31	
Non-Hispanic Black	10.57	10.51	11.26	
Other Race – including multi-racial	9.03	9.02	9.11	
Education, %				
Less than high school	13.13	12.53	20.09	<.01
High school grad/GED or equivalent	23.77	23.44	27.62	
Some college or AA degree	31.57	31.34	34.23	
College graduate or above	31.52	32.69	18.06	
Marital status, %				
Married/living with partner	31.65	32.26	24.67	<.01
Widowed/divorced/separated	9.35	8.92	14.28	
Never married	9.58	9.28	13.1	
Missing	49.42	49.55	47.95	
Alcohol user, %				
1–14 drinks in the past 12 mo	50.45	50.84	45.94	<.01
15 drinks or more in the past 12 mo	49.55	49.16	54.06	
CVD, %				
Yes	9.62	8.86	18.36	<.01
No	90.38	91.14	81.64	
Smoke, %				
Every day	13.49	12.13	29.25	<.01
Some days	4.05	3.76	7.42	
Not at all	25.98	26.1	24.7	
Missing	56.47	58.01	38.63	
Hypertension, %				
Yes	32.61	31.62	44.14	<.01
No	67.39	68.38	55.86	
Diabetes, %				
Yes	11.35	10.91	16.44	<.01
No	86.29	86.72	81.35	
Borderline	2.36	2.37	2.21	

BMI = body mass index, CVD = cardiovascular disease, HDL-C = high-density lipoprotein cholesterol, LDL = low-density lipoprotein, PIR = poverty-income ratio.

Two regression models were then fitted: Model 1 adjusted for age, sex, and race/ethnicity; Model 2 additionally adjusted for PIR, BMI, education, marital status, alcohol use, smoking, CVD, hypertension, diabetes, LDL-C, and TG.

To evaluate potential non-linear associations, we applied generalized additive models (GAMs) with restricted cubic spline regression within the weighted multivariable logistic regression framework. When the spline curve suggested a non-linear relationship, a two-piecewise logistic regression was further conducted, with the inflection point determined by maximum likelihood estimation. Subgroup analyses were performed stratified by sex, BMI categories, hypertension, and diabetes. A two-sided *P* < .05 was considered statistically significant.

## 3. Results

### 3.1. Characteristics of the participant

In the study cohort, 1088 participants (8.8%) were diagnosed with depression, while 11,222 (91.2%) were not. Table 1 presents the sample-weighted demographic characteristics. The mean age of participants was 48.01 years. Notably, no significant differences were found in age, LDL levels, race, and depression status, indicating that these factors may not independently influence the presence of depression in this sample. However, significant disparities emerged in several crucial aspects. Poverty status, HDL levels, TG concentrations, BMI, sex, educational attainment, marital status, alcohol consumption, history of CVD, smoking behavior, hypertension, and diabetes all showed statistically significant differences concerning depression.

### 3.2. Association of HDL-C with depression

Table 2 offers a synopsis of the correlation between HDL-C levels and depression. The results imply that an elevation in HDL-C levels corresponds to a decreased likelihood of depression. When considering an unadjusted model that solely took HDL-C into account, a statistically significant inverse association emerged. When contrasting participants in the uppermost and lowermost quartiles of HDL-C levels, the OR was calculated to be −0.45, with a 95% CI ranging from −0.65 to −0.25. In Model 1, after factoring in age, sex, and race, the link between HDL-C and depression grew stronger. When comparing the highest and lowest quintiles of HDL-C, the OR for depression reached −0.92, with a 95% CI spanning from −1.13 to −0.71. In Model 2, where additional covariates such as the PIR, diabetes status, and BMI were incorporated, the OR decreased to −0.09 (95% CI: −0.31 to 0.14). Moreover, there was no significant tendency indicating that higher HDL-C levels lead to a lower depression risk (*P* for trend > .05), which may suggest the presence of confounding factors.

**Table 2:** Association between HDL-C and depression.

	Unadjusted model	Model 1	Model 2
HDL-C			
Q1	Ref	Ref	Ref
Q2	−0.36 (−0.57, −0.15) 0.0008	−0.51 (−0.72, −0.30) < 0.0001	−0.15 (−0.35, 0.06) 0.1573
Q3	−0.12 (−0.33, 0.09) 0.2699	−0.43 (−0.64, −0.22) < 0.0001	0.10 (−0.11, 0.31) 0.3522
Q4	−0.45 (−0.65, −0.25) < 0.0001	−0.92 (−1.13, −0.71) < 0.0001	−0.09 (−0.31, 0.14) 0.4426
*P* for trend	<.01	<.01	.74

Model 1 adjust for: sex, age, race; Model 2 adjust for: sex, age, race, PIR, diabetes, BMI, education, marital status, alcohol, CVD, smoking, LDL, triglycerige.

BMI = body mass index, CVD = cardiovascular disease, HDL-C = high-density lipoprotein cholesterol, LDL = low-density lipoprotein, PIR = poverty-income ratio.

#### 3.2.1. Restricted cubic spline regression analysis between HDL-C and depression

**Figure 2.**
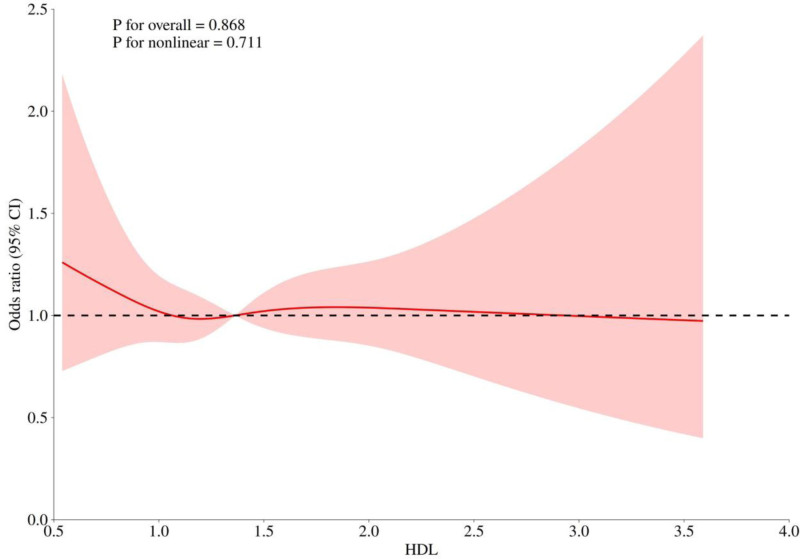
The smoothed curve-fit plot of the dose-response relationship between HDL-C and depression. HDL-C = high-density lipoprotein cholesterol.

Figure 2 depicts the non-linear link between HDL-C and depression, analyzed via smooth curve fitting and GAMs. A two-piecewise linear regression (Table 3) identified 1.09 mmol/L as the inflection point. Above this level, depression risk rose with HDL-C, yet the association was non - significant (OR = 1.12, 95% CI: 0.92–1.38, *P* > .05). Below it, higher HDL-C was linked to lower depression risk, also non - significant (OR = 0.53, 95% CI: 0.26–1.10, *P* > .05).

**Table 3:** Nonlinearity addressing of high-density lipoprotein cholesterol (HDL-C) and depression.

Depression	OR (95% CI) *P*-value
HDL-C	
Fitting model by standard logistic regression	1.03 (0.86–1.24) .7543
Fitting model by two-piecewise logistic regression	
Inflection point	1.09
<1.09	0.53 (0.26–1.10) .0908
>1.09	1.12 (0.92–1.38) .2529
*P* for log likely ratio test	.0693

Adjusted for all covariates presented in Table 2.

CI = confidence interval, HDL-C = high-density lipoprotein cholesterol, OR = odds ratio.

### 3.3. Subgroup analyses

Table 4 presents the results of the subgroup analysis, conducted to examine whether the relationship between HDL-C and depression varies across different populations and settings. The interaction test revealed a significant effect for BMI (*P* = .03), but no significant interactions were observed between HDL-C and depression with gender, hypertension, or diabetes (*P* > .05). A significant association was found in the fourth quartile (Q4) group for males (OR = 1.92; 95% CI: 0.45–3.39, *P* < .05) and non-hypertensive patients (OR = −3.64; 95% CI: −5.21 to −2.07, *P* < .05). However, no significant associations were observed for females (OR = −0.02; 95% CI: −0.35 to 0.32, *P* > .05), hypertensive patients (OR = −0.03; 95% CI: −0.39 to 0.33, *P* > .05), patients with diabetes (OR = −0.41; 95% CI: −1.01 to 0.20, *P* > .05), or those without diabetes (OR = −1.93; 95% CI: −4.17 to 0.30, *P* > .05) in Q4. Our findings suggest that the relationship between HDL-C and depression is influenced by BMI and may be particularly relevant for males and non-hypertensive individuals.

**Table 4:** Subgroup analyses stratified by sex, BMI and hypertension, diabetes.

HDL-C	Q1	Q2	*P*	Q3	*P*	Q4	*P*	*P* for interaction
Sex								
Female	Ref	−0.22 (−0.48, 0.05)	.11	−0.17 (−0.46, 0.13)	.27	−0.02 (−0.35, 0.32)	.93	.48
Male	Ref	1.88 (0.41, 3.34)	.01	1.99 (0.52, 3.45)	.01	1.92 (0.45, 3.39)	.01	
BMI (kg/m^2^)								
Underweight	Ref	0.42 (−2.72, 3.55)	.79	0.39 (−2.52, 3.30)	.79	−1.45 (−4.29, 1.38)	.31	.03
Normal	Ref	−4.62 (−11.75, 2.51)	.2	−4.40 (−11.53,2.73)	.22	4.44 (−11.57, 2.69)	.22	
Overweight	Ref	−4.07 (−11.16, 3.03)	.26	−4.34 (−11.44, 2.76)	.23	−3.91 (−11.02, 3.19)	.28	
Obese	Ref	−3.33 (−10.40, 3.75)	.36	−3.06 (−10.14, 4.02)	.40	−3.31 (−10.40, 3.77)	.36	
Hypertension								
Yes	Ref	−0.03 (−0.36, 0.30)	.85	−0.03 (−0.37, 0.31)	.86	−0.03 (−0.39, 0.33)	.88	.95
No	Ref	−3.73 (−5.29, −2.17)	<.01	−3.60 (−5.17, −2.04)	<.01	−3.64 (−5.21, −2.07)	<.01	
Diabetes								
Yes	Ref	0.14 (−0.35, 0.62)	.58	−0.33 (−0.86, 0.20)	.22	−0.41 (−1.01, 0.20)	.19	.09
No	Ref	−2.04 (−4.27, 0.20)	.07	−1.87 (−4.11, 0.36)	.10	−1.93 (−4.17, 0.30)	.09	
Borderline	Ref	4.74 (−0.73, 10.20)	.09	4.57 (−0.82, 9.96)	.10	5.84 (0.38, 11.29)	.04	

Adjusted for sex, age, race; PIR, diabetes, BMI, education, marital status, alcohol, CVD, smoking, LDL and triglycerige except the stratification factor itself. BMI was classified as underweight (BMI < 18.5), normal (18.5 ≤ BMI < 25), overweight (25 ≤ BMI < 30), and obese (BMI ≥ 30).

BMI = body mass index, CVD = cardiovascular disease, HDL-C = high-density lipoprotein cholesterol, LDL = low-density lipoprotein, PIR = poverty-income ratio.

### 3.4. Assumption diagnostics

Box-Tidwell tests suggested nonlinearity of the logit for continuous predictors (HDL-C, age, BMI, PIR, LDL, TGs), aligning with the smooth patterns reported for HDL-C (Table S1A, Supplemental Digital Content, https://links.lww.com/MD/Q615). VIFs indicated moderate-to-high collinearity among a subset of predictors: most notably BMI, age, HDL-C, and LDL (Table S1B, Supplemental Digital Content, https://links.lww.com/MD/Q615). Influence diagnostics flagged a subset of observations by residual, leverage, or Cook D criteria (Table S1C, Supplemental Digital Content, https://links.lww.com/MD/Q615); inspection did not reveal systematic data errors.

## 4. Discussion

In this cross-sectional study using NHANES data from 2015 to 2020, we identified a negative association between HDL-C and depression. A nonlinear relationship was observed, with an inflection point at 1.09 mmol/L. Subgroup analyses stratified by sex, BMI, hypertension, and diabetes revealed that the negative relationship between HDL-C and depression persisted in men and individuals without hypertension. These findings suggest that maintaining HDL-C levels slightly above the threshold could help reduce the incidence of depression.

Previous studies have found a link between HDL-C levels and depression. Zhang Cun et al^[21]^ found that patients with severe depression had lower serum HDL-C levels compared to healthy individuals. A cross-lagged panel analysis in a Chinese population of middle-aged and elderly individuals found that depression is associated with higher HDL-C levels.^[7]^ Similarly, another study found that greater depressive symptoms in adolescents were associated with higher HDL concentrations.^[14]^ In 2024, Wu Q and colleagues^[22]^ found that pregnant women with depression had higher HDL-C levels compared to pregnant women without depression. This is inconsistent with the findings of our study. Our research showed that lower HDL-C levels in American adults were negatively associated with a higher prevalence of depression. These inconsistencies with prior studies may stem from important methodological differences. For instance, Wu et al (2024) focused on pregnant women, a population with unique metabolic and hormonal alterations, whereas our analysis used a general adult population sample from NHANES.^[22]^ Similarly, Liu et al (2024) employed a cross-lagged panel design to explore bidirectional effects in middle-aged and elderly Chinese adults, while our study applied weighted multivariable logistic regression within a cross-sectional framework.^[7]^ Furthermore, studies in adolescents (e.g., Tseng et al, 2024) used different depression assessments and lipid cutoffs, which may contribute to discrepancies in observed associations.^[14]^ These variations in study design (longitudinal vs cross-sectional), population characteristics (pregnant women, adolescents, elderly vs general U.S. adults), and analytic strategies (panel analysis vs logistic regression) likely explain why our findings differ from some reports. Conversely, other studies with comparable designs and broader adult cohorts^[12,13,23]^ yielded results consistent with ours, further reinforcing the robustness of our conclusions.

To gain a more thorough understanding of the connection between HDL-C levels and depression, we employed several analytical approaches, including multiple logistic regression, stratified analyses, and trend tests. Our analysis revealed that individuals, particularly men and those without hypertension, who had lower HDL-C levels were at an increased risk for depression compared to those with higher HDL-C levels. Our findings suggest that the relationship between HDL-C and depression is influenced by BMI and may be particularly relevant for males and non-hypertensive individuals.^[24]^ The stronger association observed in men compared to women may reflect sex-specific differences in lipid metabolism and hormonal regulation. Estrogen is known to influence both HDL-C levels and inflammatory pathways, potentially buffering the relationship between HDL-C and depression in women. In contrast, men generally have lower baseline HDL-C concentrations, and small variations may have a more pronounced impact on mood regulation.^[25,26]^

Regarding hypertension, the significant association in non-hypertensive individuals but not in those with hypertension could be due to competing vascular and metabolic risks.^[27,28]^ Hypertension itself is a well-established risk factor for both CVD and depression, and its presence may overshadow or attenuate the contribution of HDL-C to depression risk.^[27]^ Conversely, in individuals without hypertension, the effect of HDL-C on depression is more readily detectable. This pattern suggests that HDL-C may exert its most relevant effects on mental health in populations not already burdened by major vascular comorbidities.

However, after accounting for various confounders such as age, sex, race, and other relevant variables, the apparent protective effect of HDL-C on depression risk was reduced. This suggests that the relationship between HDL-C and depression is likely influenced by a range of other factors. To gain a clearer and more definitive understanding, additional prospective studies are needed, particularly those that explore how gender and hypertension might modify this relationship.

Emerging evidence increasingly supports the notion that depression, from a pathophysiological standpoint, can be classified as an inflammatory disorder.^[29]^ Neuroinflammation induced by peripheral inflammation leads to both structural and functional alterations in the central nervous system, which is thought to play a key role in the development of depression.^[30]^ This process involves peripheral immune activation, which triggers the release of pro-inflammatory cytokines, resulting in behavioral, neurochemical, and neuroendocrine changes associated with depression.^[31,32]^

Although our study did not directly investigate biological mechanisms, prior literature suggests several plausible pathways through which HDL-C may be linked to depression. HDL-C has been reported to exert anti-inflammatory effects, which could theoretically mitigate neuroinflammation observed in depression.^[33,34]^ Moreover, inflammatory processes associated with depression may contribute to lower HDL-C levels, suggesting a potentially bidirectional relationship. Other studies have implicated interleukin-6–mediated inflammation, disturbances in cortisol regulation, and nutritional deficiencies resulting from appetite loss as possible contributors to the observed association between low HDL-C and depression.^[35–37]^ Taken together, these mechanisms remain speculative and require dedicated mechanistic or longitudinal studies to confirm.

This study has several limitations. First, as a cross-sectional analysis, it cannot establish causality between HDL-C levels and depression. Future prospective studies are needed to explore the underlying mechanisms linking HDL-C with depression severity. Second, due to limitations in the biochemical data available, HDL particle composition (lipoprotein particles) was not assessed in this study. Third, while the PHQ-9 is a valid and reliable tool for assessing the severity of depressive symptoms, it is not a diagnostic instrument for major depression. Longitudinal studies focusing on patients with major depression are necessary to better generalize our findings.

## 5. Conclusion

Our study identified a negative association between HDL-C levels and depression in adults, particularly among men and those without hypertension. This relationship followed a nonlinear pattern, with an inflection point at 1.09 mmol/L. We propose that lower HDL-C levels could serve as early indicators for identifying depression and may help inform treatment strategies.

## Acknowledgments

We extend our heartfelt appreciation to the staff of the National Center for Health Statistics, under the Centers for Disease Control. Their diligent work in designing, collecting, and collating NHANES data, as well as creating the public database, is highly commendable and has significantly contributed to public health research.

## Author contributions

**Data curation:** Jing Zheng, Juan Qian.

**Funding acquisition:** Minhua Li.

**Investigation:** Qi Wang.

**Project administration:** Juan Qian.

**Software:** Minhua Li.

**Writing – original draft:** Jing Zheng, Juan Qian.

**Writing – review & editing:** Qi Wang.

## Supplementary Material


