# Supplementary material for: Association between HDL-C and depression in U.S. adults: A cross-sectional analysis of NHANES
Source: Medicine (Baltimore). 2025 Nov 14;104(46):e45754. doi: 10.1097/MD.0000000000045754 (PMC12622704; doi:10.1097/MD.0000000000045754)
Supplement: Supplementary file 1 [file medi-104-e45754-s001.docx]

## Supplementary Table S1A. Box–Tidwell tests for linearity of the logit

Each continuous predictor was tested for logit-linearity by adding an interaction term x*ln(x).

| Variable | Box-Tidwell p-value | Linearity_OK_(p>0.05) |
| --- | --- | --- |
| Age | 0.021791 | FALSE |
| BMI | 0.919218 | TRUE |
| HDL | 0.977675 | TRUE |
| LDL | 0.847474 | TRUE |
| PIR | 0.566051 | TRUE |
| Triglyceride | 0.561153 | TRUE |

## Supplementary Table S1B. Variance Inflation Factors (VIFs) for predictors

VIF quantifies multicollinearity among predictors; values >10 typically indicate problematic collinearity.

| Predictor | VIF |
| --- | --- |
| BMI | 17.385 |
| Age | 16.875 |
| HDL | 13.568 |
| LDL | 10.116 |
| C(Educationlevel)[T.4.0] | 9.725 |
| C(Educationlevel)[T.3.0] | 6.556 |
| C(Educationlevel)[T.5.0] | 5.846 |
| C(Race)[T.3] | 5.552 |
| PIR | 5.306 |
| Triglyceride | 4.958 |
| C(Race)[T.4] | 4.077 |
| C(Smoke)[T.3.0] | 3.42 |
| C(Educationlevel)[T.2.0] | 3.254 |
| C(Race)[T.5] | 2.417 |
| C(Sex)[T.2] | 2.149 |
| C(highbloodpressure)[T.1.0] | 2.117 |
| C(alcohol)[T.2.0] | 2.104 |
| C(Race)[T.2] | 1.688 |
| C(alcohol)[T.3.0] | 1.656 |
| C(Marital_status)[T.2.0] | 1.645 |
| C(diabetes)[T.1.0] | 1.551 |
| C(Marital_status)[T.3.0] | 1.529 |
| C(alcohol)[T.4.0] | 1.512 |
| C(Smoke)[T.2.0] | 1.48 |
| C(myocardialinfarction)[T.1.0] | 1.399 |
| C(alcohol)[T.6.0] | 1.31 |
| C(heartfailure)[T.1.0] | 1.308 |
| C(alcohol)[T.5.0] | 1.291 |
| C(anginaanginapectoris)[T.1.0] | 1.174 |
| C(alcohol)[T.8.0] | 1.15 |
| C(stoke)[T.1.0] | 1.12 |
| C(alcohol)[T.10.0] | 1.117 |
| C(alcohol)[T.12.0] | 1.074 |
| C(alcohol)[T.15.0] | 1.063 |
| C(alcohol)[T.7.0] | 1.062 |
| C(alcohol)[T.9.0] | 1.032 |
| C(alcohol)[T.13.0] | 1.03 |

## Supplementary Table S1C. Influence diagnostics for the logistic regression model (weights normalized)

Standardized residuals, leverage, and Cook’s distance were computed from a weighted GLM with survey weights normalized (mean=1) to stabilize influence statistics.

| Metric | Value |
| --- | --- |
| N | 1001.0 |
| p_(parameters) | 46.0 |
| StdResid_\|>3\|_count | 36.0 |
| High_Leverage_count | 113.0 |
| High_CooksD_count | 73.0 |
| Any_Flagged_count | 154.0 |
| Leverage_threshold | 0.0919080919080919 |
| CooksD_threshold | 0.0039960039960039 |
| Max_CooksD | 372940274925309.1 |
